# Analysis of population genetic structure of Indian *Anopheles culicifacies* species A using microsatellite markers

**DOI:** 10.1186/1756-3305-6-166

**Published:** 2013-06-06

**Authors:** Sujatha Sunil, Om P Singh, Nutan Nanda, Kamaraju Raghavendra, BP Niranjan Reddy, Sarala K Subbarao

**Affiliations:** 1 National Institute of Malaria Research, Sector 8, Dwarka, New Delhi, 110 077, India; 2 Present address: International Centre for Genetic Engineering and Biotechnology, New Delhi, 110 067, India; 3 Present address: Indian Council of Medical Research, Ansari Nagar, New Delhi, 110 029, India

**Keywords:** *Anopheles culicifacies* complex, Microsatellite markers, Population genetics, Hardy-Weinberg Equilibrium

## Abstract

**Background:**

*Anopheles culicifacies sensu lato* is an important vector of malaria in Southeast Asia contributing to almost 70% of malaria cases in India. It exists as morphologically similar sibling species A, B, C, D and E with varied geographical distribution patterns. Vector control measures have been difficult for this important vector as the sibling species have developed varying levels of resistance to the currently used insecticides. In view of the importance of this vector, we developed and validated a set of microsatellite markers and the same were used to analyze the population genetic structure of five different geographical populations of *An*. *culicifacies* A.

**Methods:**

*Anopheles culicifacies* A samples were collected from different localities across India, and genotyping was performed using eight microsatellite markers on ABI Prism 310 Genetic Analyzer. Several statistical analyses were performed to ascertain the genetic diversity that exists within and between the populations.

**Results:**

The markers were found to be moderately polymorphic in the populations. Genetic analysis indicated significant genetic differentiation between the majority of the population pairs analyzed and was not found to be related to the geographical distances between populations.

**Conclusion:**

This is the first and successful attempt to test the microsatellite markers developed for population genetic analysis of *An*. *culicifacies* A. Host feeding and breeding habits of species A suggest that factors other than ecological and geographical barriers were responsible for the genetic differentiation that has been observed between the populations.

## Background

*Anopheles culicifacies sensu lato* has a wide distribution in India and extends into the west to Ethiopia, Yemen, Iran, Afghanistan and Pakistan, and in the east to Bangladesh, Myanmar, Thailand, Cambodia and Vietnam and is also found in Nepal and southern China in the north and extends to Sri Lanka in the south [1,2]. It is an important malaria vector in India, Sri Lanka and in the countries west of India. It is responsible for 60-70% of new malaria cases in India [2]. This taxon has now been recognized as a species complex with five members provisionally designated as species A and B [3], C [4], D [5] and E [6]. Species, A, B, C and D, were recognized following the observation of a total absence or significant deficiency of heterozygotes in natural populations for the alternate arrangements observed in polytene chromosomes due to paracentric inversions. The fifth species, species E, was identified by correlating Y-chromosome polymorphisms of sons and the sporozoite positivity of mothers.

There is little information available about the population structure and gene flow that occurs between and/or within *An*. *culicifacies* sibling species populations in India. Several studies were carried out to examine the population structure of vectors in Africa and other countries, namely *An*. *gambiae s*. *s*. [7], *An*. *arabiensis*[8], *An*. *funestus*[9], *An*. *darlingii*[10] and many other vectors, using microsatellite markers and mitochondrial genes. Recently, two major vectors found in India, *An*. *stephensi* an urban malaria vector [11] and *An*. *baimaii*, a vector in north eastern states [12] were analysed for population genetic structure and gene flow using microsatellite markers and mitochondrial DNA genes respectively. Microsatellites are highly polymorphic genetic markers used extensively for studying genetic structure of populations. Realizing the importance of knowledge on the population structure and gene flow for implementing insecticide resistance management strategies for the control of *An*. *culicifacies* sibling species, microsatellite markers developed in our laboratory [13] were used in this study to understand the population structure of *An*. *culicifacies* species A populations, and the results are reported in this paper.

## Methods

### Sample collection and species identification

**Figure 1:**
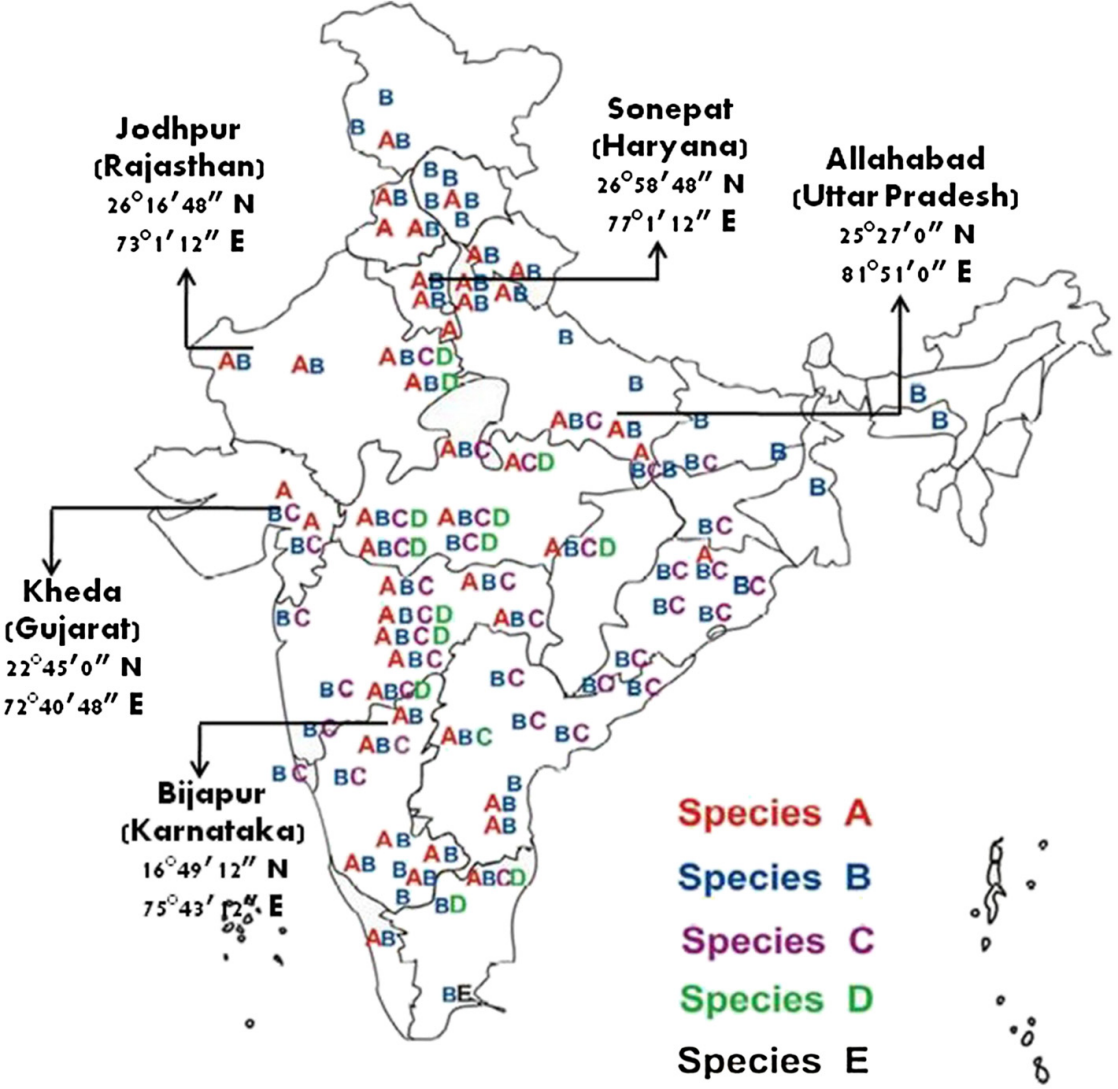
**Distribution of *An*. *culicifacies *sibling species in India and sites used in the study (source Ref [**[24]**]).** Map of India showing the distribution of *An*. *culicifacies* sibling species in India. Site locations are indicated by arrows. States are indicated within parenthesis.

*Anopheles culicifacies* samples were collected from five different localities in India, namely the states (districts); Haryana (Sonepat), Gujarat (Kheda), Karnataka (Bijapur), Rajasthan (Jodhpur), and Uttar Pradesh (Allahabad). Specific spatial details of the collection sites are given in Figure 1[14] and were selected to represent different eco-settings that prevail in India. Collections of indoor-resting mosquitoes were made by hand-collection using suction tube and torchlight. Specimens collected from resting sites were confirmed as *An*. *culicifacies* on a morphological basis, following the mosquito identification key by Christophers [15]. For sibling species identification, semi-gravid females were used for cytotaxonomic identification and genotyping. Ovaries from individual semi-gravid females were pulled out and stored in modified Carnoy’s fixative (1:3 glacial acetic acid and methanol) and the carcasses of the mosquito were stored in isopropanol for DNA isolation. The two vials of each mosquito were given a corresponding code number for species correlation. Ovaries were processed for the preparation of polytene chromosomes and diagnostic inversion genotype analyses [16] were used for sibling species identification. In cases where specimens were not in a suitable stage for cytological identification, 28S-D3 – PCR assay was used [17].

### Study sites

Study sites are shown in Figure 1[14], which also shows the distribution of sibling species mapped from earlier studies based on which the present study sites were selected.

### Genomic DNA extraction and Microsatellite genotyping of field collected samples

The DNA was isolated from individual mosquitoes following the procedure described previously [18] and stored at −20°C until further use. In total thirty-one microsatellite markers were isolated in *An*. *culicifacies A* in our previous investigation [13]. Randomly, eight microsatellite markers, which were found to be polymorphic in the laboratory reared specimens and in one field population of *An*. *culicifacies* were used in the present study. Linkage relationship of these loci is not known. Each of the primer pairs belonging to the selected loci (Table 1), were labeled at 5′ with TET™, HEX™ or 6-FAM™ fluorescent dyes (Microsynth Corporation, Switzerland). Multiplex PCRs were set up by grouping 2 or 3 primer pairs together depending on their annealing temperatures, dyes, and sizes and PCR amplifications. The PCR primers and procedures followed in the study were same as described earlier [13]. The resultant amplified products were resolved using an ABI Prism 310 Genetic Analyzer (Applied Bio systems). Alleles were sized relative to an internal fluorescent standard marker and the results were analyzed using the Genescan software version 3.1 (Applied Bio systems).

**Table 1:** List of microsatellite markers used in the study with details of the primer sequences

**S. No**	**Microsatellite markers**	**Primer sequence 5′-3′**
1	AcA11B5	CGGAAAACGTTGCAACAAAATC
		ATCCAACCGTAGCCATAACAAAAC
2	AcA59	GCGTAGGTCAACCGTAATGC
		TCCCACATACCGATACACCA
3	AcAVB93	GTCCTTTGCAAATCACATCGG
		TTAATGACTTCAATCCACAAACCC
4	AcAVIIB46	AACCGGAAGCAGTATCGCACAC
		GAGGCTCCTTCGTTCCATCCG
5	AcAVIB213	ATAAAACGCCCCGCATCATACATG
		ACGGCACATTCCCTCCCATAG
6	AcA61	CGAATGCATTTCGCTTGATA
		TTCCTACTCACCAGCTGAAC
7	AcAVB93A	GTGGCCGTTGTTCGTCCTTTTG
		TGCTCGTGGCGTTCGTGAGTC
8	AcAVIIIB40	TCAAGCTGGACAATGTAACTCAAC
		TTCAATCAAACCCAGCCAAAC

### Statistical analysis

Random mating i.e., agreement to Hardy-Weinberg Equilibrium (HWE) within each of the populations was tested by using the Arlequin v.2.0 software [19]. An unbiased estimate of the exact *P*-value for each locus was computed using the Markov chain method of Geo and Thompson [20] with the forecasted chain length of 100,000 steps and dememorization steps of 1000. The observed proportion of heterozygote deficiencies (D) and the frequencies of null alleles (r) that caused the heterozygote deficiencies were estimated following the method described in Chakraborty *et al*., [21]. For calculation of D and r, the following equations were used: D = (H_E_-H_o_)/H_E_ and r = (H_E_-H_o_)/(H_E_ + H_o_); where H_E_ is expected heterozygote frequency and H_O_ is observed frequency of heterozygotes.

Genetic variability among the populations was measured by Wright’s F-statistics [22]. Further, F-statistics values were also calculated according to the method of Weir and Cockerham using the GenAlex v.5.4 (MS Excel-based genetic analysis tool) [23]. The significance of F_ST_ values was tested using the formula described by Workman and Niswander where Chi square = 2NF_ST_ (where N is the population size with n-1 degrees of freedom for ‘n’ subpopulations) [24].

The effective migration rate (Nm) was estimated according to Slatkin’s (1987) formula; Nm = 1/4 [(1/ F_ST_)-1] [25]. To investigate, if levels of differentiation were related to geographical distances, the regression of F_ST_ (1-F_ST_) on the natural log (*ln*) of geographical distance was used [26].

## Results

Morphologically identified *An*. *culicifacies* specimens were collected from five different localities in India that were selected to best represent the diversity of Indian geography and its ecology (Figure 1). Of the specimens identified, both cytotaxonomically and by PCR methods, a total of 104 species A samples were analyzed for the population structure of *An*. *culicifacies* in India. Details of the number of samples from each of the five localities are given in Table 1 and the location of the study sites are shown in Figure 1.

### Distribution and the level of genetic diversity

A total of 104 samples were analyzed for eight microsatellite markers, in order to understand various population genetic parameters that might be influencing the present genetic make-up of *An*. *culicifacies*. Genetic diversity using microsatellite markers was compared by examining the number of alleles and its heterozygosity observed among the five different populations of species A. Various population parameters such as the number of samples from each of the five locations, total number of alleles observed (N), expected and observed heterozygosity (H_o_, H_e_), deficiency of number of heterozygotes (D), etc. were analyzed (Table 2). Among the populations examined, a total of 69 alleles were observed among the eight loci. The observed loci were found to be moderately polymorphic among all the populations. In all the five populations of species A, the maximum number of alleles observed ranged between 4 and 17. Of the eight microsatellite loci, *ACAV1B213* was found to be highly polymorphic with 17 alleles. This marker had a maximum of 12 alleles in Sonepat and Kheda populations, while 8, 9, and 11 alleles were observed in Bijapur, Allahabad, and Jodhpur, respectively. The AcAVIIB46 and AcAVB93A loci were least polymorphic with the maximum number of alleles being four. The four alleles of AcAVIIB46 were observed in Bijapur and Kheda populations while AcAVB93A displayed four alleles only in Bijapur. The weighted average of number of alleles between the eight markers among all the samples ranged from 2.5 to 10.6, while between the five different populations the range of average alleles varied from 4.41-5.74. Among the five populations of species A, the average of expected heterozygosity ranged from 0.42 - 0.87 per locus suggesting that expected heterozygosity does not significantly vary between the populations to the average of the total expected heterozygosity observed cumulatively in the total population. The observed heterozygosity among the eight loci ranged from 0.199 to 0.418 but did not differ significantly among the populations (0.182 to 0.4706) (F-0.53, d.f.-6, P > 0.05).

**Table 2:** Details of genetic diversity at microsatellite loci among five populations of species A

**Microsatellite markers (total no. of alleles observed)**	**Genetic diversity**	***An. culicifacies *species A**					**Weighted average**
		**Allahabad**	**Bijapur**	**Sonepat**	**Kheda**	**Jodhpur**	
***ACA11B5*** (**9**)	**N** (**103**)	23	24	28	17	11	
	**N**_**a**_	3	4	8	5	3	**4**.**9223**
	**H**_**o**_	0.34783	0.45833	0.39286	0.58824	0.27273	**0**.**4175**
	**H**_**E**_	0.50338	0.7039	0.73896	0.68806	0.60606	**0**.**6556**
	**D**	0.294**	0.304*	0.45***	0.1192	0.4803*	**0**.**3289**
	**r**	0.1721	0.1795	0.2879	0.0634	0.3161	**0**.**2027**
***ACA59*** (**8**)	**N** (**87**)	13	23	27	16	8	
	**N**_**a**_	5	4	3	6	3	**4**.**1149**
	**H**_**o**_	0.30769	0.21739	0.37037	0.25	0.25	**0**.**2874**
	**H**_**E**_	0.51385	0.30628	0.45283	0.39315	0.675	**0**.**4327**
	**D**	0.377**	0.2744	0.1	0.2471	0.579**	**0**.**2586**
	**r**	0.2325	0.159	0.0526	0.1409	0.4074	**0**.**1565**
***ACAVB93***(**9**)	**N** (**88**)	17	21	28	15	7	
	**N**_**a**_	8	5	5	8	6	**6**.**1705**
	**H**_**o**_	0.52941	0.57143	0.57143	0.53383	0.14286	**0**.**5228**
	**H**_**E**_	0.78431	0.63415	0.50325	0.8	0.95604	**0**.**6754**
	**D**	0.29***	0.0345	−0.1561	0.3084*	0.82***	**0**.**1342**
	**r**	0.1752	0.0175	−0.0724	0.1823	0.6989	**0**.**1017**
***ACAV11B46***(**4**)	**N** (**100**)	20	25	28	17	10	
	**N**_**a**_	3	4	3	4	2	**3**.**32**
	**H**_**o**_	0	0.24	0.17857	0.41176	0.2	**0**.**199**
	**H**_**E**_	0.23718	0.4751	0.45974	0.57041	0.27895	**0**.**4198**
	**D**	1***	0.452**	0.5725	0.204*	−0.1111	**0**.**4968**
	**r**	1	0.2916	0.4011	0.1136	−0.0526	**0**.**3993**
***ACAV1B213***(**17**)	**N** (**94**)	21	23	22	17	11	
	**N**_**a**_	9	11	12	12	8	**10**.**617**
	**H**_**o**_	0.2381	0.26087	0.39286	0.47059	0.18182	**0**.**321**
	**H**_**E**_	0.91057	0.9256	0.73896	0.94118	0.90909	**0**.**8713**
	**D**	0.72***	0.70***	0.46***	0.47***	0.78***	**0**.**6052**
	**r**	0.5652	0.5434	0.2943	0.3061	0.6349	**0**.**4468**
***ACA61*** (**10**)	**N** (**98**)	23	20	28	17	10	
	**N**_**a**_	7	8	6	6	5	**6**.**508**
	**H**_**o**_	0.17391	0.2	0.53571	0.41176	0.4	**0**.**3469**
	**H**_**E**_	0.75169	0.88077	0.84351	0.81462	0.76842	**0**.**8169**
	**D**	0.75***	0.76***	0.34**	0.46**	0.41***	**0**.**5498**
	**R**	0.6052	0.6126	0.2034	0.2979	0.2558	**0**.**0015**
***ACAVB93A*** (**4**)	**N** (**100**)	20	25	28	16	11	
	**N**_**a**_	2	4	2	2	2	**2**.**5**
	**H**_**o**_	0	0.04	0	0.0625	0	**0**.**02**
	**H**_**E**_	0.23333	0.46286	0.54156	0.46164	0.50216	**0**.**4431**
	**D**	1**	0.9***	1***	0.85**	1**	
	**r**	1	0.8249	1	0.7322	1	**0**.**9134**
***ACAV111B40*** (**8**)	**N** (**98**)	23	22	28	17	8	
	**N**_**a**_	4	5	3	3	7	**4**.**0102**
	**H**_**o**_	0.21739	0.31878	0.32143	0.35294	0.625	**0**.**3267**
	**H**_**E**_	0.24444	0.59725	0.43377	0.54367	0.90833	**0**.**4838**
	**D**	−0.0798	0.454**	0.2455	0.2842	0.2453*	**0**.**2226**
	**r**	−0.0384	0.2936	0.1399	0.1656	0.1398	**0**.**137**
**Total**	**N**(**678**)						
	**N**_**a**_	5.08	5.55	5.07	5.74	4.41	**5**.**17**
	**H**_**o**_	0.2188	0.2842	0.3439	0.3864	0.25	**0**.**2967**
	**H**_**E**_	0.5189	0.6161	0.5856	0.6527	0.6845	**0**.**6116**
	**D**	0.5463	0.4896	0.3743	0.3652	0.5282	**0**.**4607**
	**r**	0.4679	0.3686	0.2893	0.2486	0.4311	**0**.**3611**

( ) - Number in parenthesis for N indicates total number of *An*. *culicifacies* specimens analyzed, D- observed proportional heterozygote deficiencies = (H_E_-H_o_)/H_E,_ r - frequency of null alleles = (H_E_-H_o_)/(H_E_ + H_o_) = D/2-D, N- Number of samples, N_a_- Number of alleles, H_o_- Observed heterozygosity, H_E –_ Expected heterozygosity, *P < 0.05 > 0.01; **P < 0.01 > 0.001; ***P < 0.001.

### Hardy-Weinberg equilibrium (HWE)

The genotype frequencies at several loci did not conform to HWE. For the majority of markers, the observed number of heterozygotes was less than the expected number and this deficiency was statistically significant (P < 0.05). A total of 40 tests (8 loci, 5 populations) in species A for conformance to HWE at the locus level within the populations were performed. Of these tests, 29 (72.5%) tests showed a significant deviation from HWE in species A. Furthermore, in all the cases heterozygote deficiency was observed (Table 2). The loci ACAV1B213, ACA61 and ACAVB93A showed a significant heterozygote deficiency in all the five populations, while the locus ACA11B5 showed a significant departure in four out of the five populations. Loci AcAB93 and AcAVIIB46 showed heterozygote deficiency in three populations while loci AcA59 and AcAVIIIB40 showed heterozygote deficiency in only two populations. Presence and frequency of null alleles was tested using the method of Chakraborty *et al*. [21] (Table 3). Average estimate of null allele frequency ranged from 0.02 to 1 with a maximum average of 0.45 in all the populations. Locus AcAVB93A showed the maximum frequency of null alleles in all the populations, while ACA11B5 contributed the least (Table 3).

**Table 3:** **Number of alleles observed for each locus**, **and the allele with the maximum frequency observed in the population of *An***. ***culicifacies *species A**

**Microsatellite markers**	***ACA11B5***	***ACA59***	***ACAVB93***	***ACAVIIB46***	***ACAVIB213***	***ACA61***	***ACAVB93A***	***ACAVIIIB40***
Total number of alleles observed	7	7 + 1*	9	4	17	10	4	8
Populations	Alleles with maximum frequency (allelic frequency)							
Allahabad (Uttar Pradesh) N = 23	104* (0.68)	206 (0.69)	140 (0.44)	134 (0.9)	100 (0.21)	200 (0.47)	110 (0.9)	112 (0.9)
Bijapur (Karnataka) N = 24	106 (0.4)	206 (0.84)	140 (0.6)	134 (0.72)	118 (0.2)	200 (0.25)	110 (0.75)	114 (0.48)
Sonepat (Haryana) N = 28	106 (0.45)	206 (0.74)	140 (0.68)	137 (0.72)	100 (0.35)	198 (0.27)	112 (0.55)	114 (0.72)
Kheda (Gujarat) N = 17	106 (0.44)	206 (0.81)	140 (0.38)	137 (0.66)	112 (0.24)	200 (0.39)	110 (0.74)	114 (0.62)
Jodhpur (Rajasthan) N = 11	104 (0.64)	206 (0.5)	130 (0.29)	134 (0.9)	112(0.26) 114 (0.26)	200 (0.5)	108 (0.74)	106 (0.26)

* Allele that had maximum frequency, Number in parenthesis is the frequency of the allele observed in the population.

### Correlation between the population divergence and isolation of populations by distance

The five populations exhibited remarkably similar allelic distributions on averaging all loci but differential patterns were observed among the loci in four out of eight loci (Table 3). A single allele of 206 bp of the marker ACA59 was predominant in all the five populations of species A, and with reference to the ACAVIB213 locus, each of the populations had a different predominant allele (Table 3). There were also alleles unique to one or the other populations, but the frequencies of these alleles were very low (data not shown). The maximum frequency of alleles at each of the loci ranged from 0.2 to 0.9 in the populations (Table 3).

**Figure 2:**
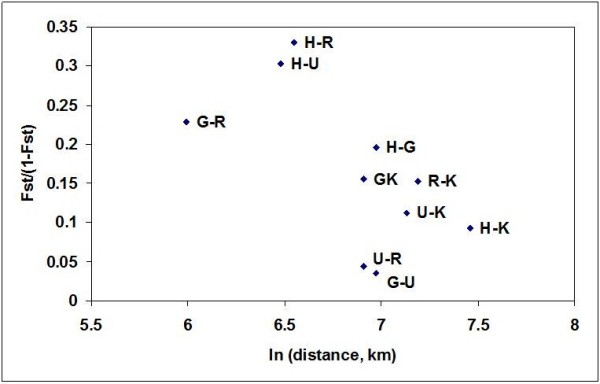
**Correlation between F**_**ST**_**/(1-F**_**ST**_**) of population pairs and geographical distances of *An. culicifacies *species A.** H- Haryana, R-Rajasthan, G-Gujarat, K-Karnataka, U-Uttar Pradesh.

**Figure 3:**
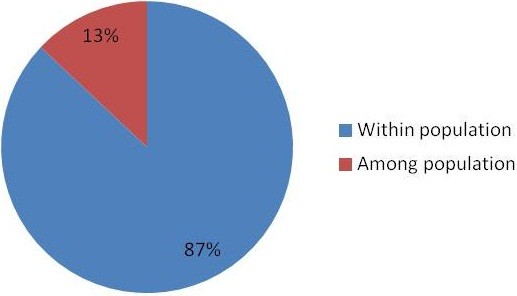
**Analysis of molecular variance based on Fst values calculated for five *An*. *culicifacies *species a population.**

Genetic variability between species A populations was studied using F-statistics. The pair-wise genetic differentiation among species A populations were found to be significant in eight out of the ten combinations and ranged from 0.0853 – 0.2483. The populations sampled from five different geographical regions represented northern, western, eastern, and southern parts of the country (Figure 1). The shortest distance between the two populations was 400 km between the districts Kheda and Jodhpur, while the longest was 1730 km between the districts Sonepat and Bijapur. A high level of genetic differentiation was observed between the species A populations. The mean F_ST_ for all the markers and all the populations was 0.155. The F_ST_ values were non-significant for two population comparisons, namely Kheda and Sonepat; and Bijapur and Sonepat, which indicates a low genetic differentiation between these populations. For all other population comparisons, the values were significant suggesting a high genetic differentiation. Furthermore, to understand the role of geographical distance in generating the genetic distance between the sampled populations, the Mantel test was performed. The test revealed no significant correlation between the pair-wise F_ST_/(1 - F_ST_) against the natural logarithm of pair-wise geographical distance (r2 = 0.3952; Figure 2), suggesting that the population genetic structure of *An*. *culicifacies* in India did not conform to the isolation-by-distance model. This was also evident from the analysis of molecular variance which is calculated based on the F_ST_ values, and is found to be greater within the pairs of populations analyzed (87%) than that among the populations (13%) (Figure 3).

### Gene flow

*The Nm*-values were calculated based on the F_ST_ values for all the comparisons and the values are tabulated in Table 4. In all the pair-wise population combinations, except for two comparisons (Sonepat vs. Allahabad; and Jodhpur vs. Sonepat), Nm values were >1 with the maximum of 7.6 between Sonepat and Kheda. The observation of low Nm values suggests the greater genetic differentiation between the populations. Thus, from the analysis of Nm values, it appears that maximum differentiation was in Allahabad vs. Sonepat comparisons (Nm = 0.83) and Jodhpur vs. Sonepat (Nm = 0.70). The two comparisons with maximum Nm values observed in this study are Kheda vs. Sonepat (7.6), and Kheda vs. Bijapur (5.6) which are distantly located, at 1000 and 1400 km apart, respectively, and it is only for these two population comparisons, that the _FST_ values are non- significant. The measure of gene flow again supports that the genetic differentiation observed is not related to geographical distance.

**Table 4:** **F**_**ST **_**and *Nm *values for the population comparisons of *An***. ***culicifacies *populations**

	**U.P.-A**	**Kar-A**	**Har-A**	**Guj-A**	**Raj-A**
**U.P.-A**	0	2.22	0.83	1.27	1.61
**Kar-A**	0.10111*	0	2.68	5.60	1.64
**Har-A**	0.232159**	0.085316*	0	7.06	0.76
**Guj-A**	0.164071**	0.042759 NS.	0.034211 NS.	0	1.10
**Raj-A**	0.134222*	0.132353*	0.248338**	0.185699*	0

*NS* Non-significant; *P < 0.01; **P < 0.001.

Figures above diagonal are Nm values and below diagonal are F_ST._

## Discussion

All the eight microsatellite markers examined in *An*. *culicifacies* species A populations were polymorphic. Maximum number of alleles at each locus ranged from 4 to 10 with the exception for one marker (ACAVVIB213), which exhibited 17 alleles, suggesting that these markers are moderately polymorphic in the populations. Except for AcAVIIB46 and AcAVB93A with the least number of alleles, the other markers did not exhibit all the alleles in any of the five populations. Significant deviations from the Hardy-Weinberg expectations for these markers were observed in the populations studied. The deficiency of heterozygotes observed in these populations could be due to population sub-structuring or due to the presence of null alleles, *i*.*e*. mutation/s in the primer sites that may prevent annealing of primers to the DNA samples. This results in either total failure of amplification (homozygous for null allele), or most commonly one strand will be amplified and the sample will be scored as a homozygote, which in fact is a heterozygote [27]. During genotyping, a few DNA samples did not amplify for one or the other marker. In order to rule out the problems of multiplexing, these samples were also genotyped separately by setting up an individual PCR. As these samples were normally amplified in some other combinations, non-amplification is necessarily not due to the poor quality of DNA or other general PCR procedural problems. Grouping together of different gene pools (Wahlund effect) and non-random mating within the populations i.e. presence of sub-populations as a reason for the deficiencies is not being favored because (i) collections were of only indoor-resting mosquitoes, collected from both human dwellings and cattle sheds, and mosquitoes were collected from structures within a single village or one or two more which are within 5–10 km range, (ii) the extensive cytotaxonomic studies carried out in different parts of the country did not indicate any sub-populations and (iii) that D values up to 10% do not indicate the presence of sub-populations [20]. The D-values observed in this study are significantly low. Therefore, presence of null alleles is being considered to be mainly responsible for the deficiencies of heterozygotes observed at these loci. However, it is not known whether the markers not being in Hardy-Weinberg equilibrium has had any influence on the population genetic parameters estimated in this study. Keeping this in view and that the markers were randomly selected not knowing whether they represent the entire genome, a few conservative conclusions are being drawn from the F-statistics data. F_ST_ values have shown a great genetic differentiation between the pairs of populations analyzed. Low Nm values (<1) between Haryana and Uttar Pradesh, and Rajasthan and Haryana suggest a limited gene flow. The geographical distances of these two population pairs were less than those observed for Gujarat and Haryana, and Gujarat and Karnataka which had maximum Nm values (>5 and 7 respectively).

Vindhyachal Mountain ranges which pass through central India (Maharashtra and Madhya Pradesh States) separate northern and southern parts of India. Karnataka is located in the southern part of India. There are no known geographical barriers that exist between the other four populations studied. Furthermore, anopheline species in general are known to have limited flight range. For species A, which is predominantly zoophagic (maximum anthropophilic index observed was 3-4%), *An*. *culicifacies* being a rural mosquito and agriculture being practiced extensively in these areas indicate free availability of cattle for blood feeding. Irrigation channels are preferred breeding habitat for species A. This suggests that other than geographical or ecological, some other barriers are playing a role for differential genetic differentiation levels observed between these populations. Residual sprays with effective insecticides alter and interfere with population sizes. At the time of collection of mosquitoes, no spray operations were going on in sites in Uttar Pradesh, Haryana and Rajasthan states. In Gujarat and Karnataka, malathion was being sprayed in the study sites, which even at present is effective on *An*. *culicifacies* populations in these areas. Therefore, the effect of insecticide sprays on the mosquito population sampling cannot be ruled out.

## Conclusion

This is the first and successful attempt made to study the population genetic structure of *An*. *culicifacies* using microsatellite markers. Genetic analysis of five different populations of species A was carried out with eight microsatellite markers. F_ST_ values indicated significant genetic differentiation between the majorities of the population pairs analyzed. We hope that these results may add a further step in understanding the dynamics of the vector species for planning effective vector control activities based on population genetic structure.

## Competing interests

The authors declare they have no competing interests.

## Authors’ contributions

SKS conceived and RK, OPS and SKS planned the study. RK made the field collections and NN identified all mosquito samples used in study. SS was involved in setting up all experiments. OPS and NR performed data analysis. SS, NR and SKS drafted the manuscript. All authors read and approved the final version of the manuscript.
